# Regarding the Zoonotic Transmission of *Giardia duodenalis* Infection

**DOI:** 10.3390/ani16152382

**Published:** 2026-08-03

**Authors:** Luis Fonte, María Ginori, Yamilé Aleaga, Enrique J. Calderón, Yaxsier de Armas

**Affiliations:** 1 Centro de Investigación, Diagnóstico y Referencia, Instituto de Medicina Tropical “Pedro Kourí”, La Habana 11400, Cuba; 2 Departamento de Docencia, Policlínico Universitario “Plaza de La Revolución”, La Habana 10609, Cuba; ginorigilke@gmail.com; 3 Departamento de Microbiología Clínica, Instituto de Medicina Tropical Pedro Kourí, La Habana 11400, Cuba; yamile.aleaga@ipk.sld.cu; 4 Instituto de Biomedicina de Sevilla, Hospital Universitario Virgen del Rocío/Consejo Superior de Investigaciones Científicas/Universidad de Sevilla, 41013 Sevilla, Spain; ecalderon@us.es; 5 Centro de Investigación Biomédica en Red de Epidemiología y Salud Pública (CIBERESP), 28029 Madrid, Spain; 6 Departamento de Microbiología y Patología, Centro Universitario de Ciencias de la Salud, Universidad de Guadalajara, Guadalajara 44100, Mexico; 7 Departamento de Anatomía Patológica, Instituto de Medicina Tropical “Pedro Kourí”, La Habana 11400, Cuba

**Keywords:** *Giardia duodenalis*, giardiasis, zoonosis, zoonotic transmission, epidemiological evidence, genetic evidence

## Abstract

*Giardia duodenalis*, a member of the genus *Giardia*, is a common intestinal protozoan parasite in humans and some farm, companion, and wild animals. Due to the uniform morphology of the parasites sampled from a wide range of hosts, the taxonomy of the genus *Giardia* has been the subject of debate for more than a century. The confusion generated by this controversy, and the technological limitations to carry out the necessary research to clarify the matter, led to an insufficient understanding of the epidemiology of the infection, mainly in aspects related to host specificity and zoonotic transmission. The results of molecular and epidemiological studies conducted over the last four decades, which are reviewed in this paper, allow us to conclude that zoonotic transmission of *Giardia duodenalis* infection is possible. Concurrently, these findings suggest that most *Giardia* infections are host-specific, rendering zoonotic transmission relatively uncommon. Nevertheless, from a public health perspective, such transmission remains a critical concern for vulnerable groups, particularly children and immunocompromised individuals. In these populations, even infrequent zoonotic events can lead to severe clinical outcomes, including chronic diarrhea, malabsorption, and failure to thrive.

## Simple Summary

This work addresses the long-standing uncertainty regarding whether the microscopic parasite *Giardia duodenalis* can be transmitted from animals to humans. This review evaluates forty years of genetic and epidemiological tracking data to clarify the actual frequency and risk of animal-to-human transmission. The results show that while zoonotic transmission is biologically possible and poses a risk to vulnerable groups, such as children, it is relatively uncommon. Modern molecular typing reveals that most strains of this parasite are highly host-specific, predominantly restricted to distinct animal groups. Therefore, human-to-human contact and contaminated water drive the vast majority of human infections. These findings help public health officials design more effective disease control strategies. Rather than overemphasizing the management of wild or domestic animals, communities can better protect public health by prioritizing cleaner drinking water, safer sewage treatment, and improved personal hygiene.

## 1. Introduction

*Giardia duodenalis* (syn. *G. lamblia*, *G. intestinalis*) is one of the species that form part of the genus *Giardia* [1]. The infection by *G. duodenalis*, known as giardiasis, is cosmopolitan [1,2,3]. However, giardiasis prevalences are higher in low- and middle-income countries than in high-income ones, ranging from 8.0 to 30.0% in the former, and from 0.4 to 7.5% in the latter [4]. Globally, an estimated 184 million giardiasis cases occur each year [5]. In addition, an increasing number of giardiasis outbreaks have been reported since 1954 [6,7].

*Giardia duodenalis* life cycle consists of two stages: trophozoite, the vegetative form, which replicates and exerts pathogenic effects in the host’s small intestine; and cysts, the environmentally stable phase, which are released in feces and transmitted via the fecal-oral route [1]. The clinical manifestations of giardiasis include abdominal pain, watery diarrhea, vomiting, and extraintestinal symptoms, such as urticaria and arthritis [3,8]. In addition, giardiasis can lead to malnutrition and weight loss, particularly in children in low- and middle-income countries, with the long-term consequences of growth and cognitive development impairment [9].

This parasite has been found infecting farm, companion, and wild animals, which has led to the longstanding concern regarding the zoonotic transmission of the infection [1]. In 1979, the then unquestioned nonspecificity of *G. duodenalis* hosts and the reports of possible contamination of water sources by animals parasitized by this protozoan prompted the WHO, possibly somewhat prematurely, to categorize this species as a zoonotic parasite [10]. Since then, the search for evidence to confirm the zoonotic transmission of this parasitosis has been intense. The accumulated information, especially the finding of epidemiological and genetic data in favor of the transmission between companion animals and humans, has made it possible to conclude that, indeed, giardiasis is a zoonosis [1,2,11,12,13]. However, most *Giardia* infections are host-specific and, consequently, the frequency of zoonotic transmission events appears to be low [11,12,13]. Here, we comment on the current evidence and the true extent of the zoonotic transmission of *G. duodenalis* infection.

Literature Search and Selection Strategy: A review and analysis of molecular and epidemiological studies published over the last four decades was carried out. The search focused on research documenting host specificity and evidence of zoonotic transmission of giardiasis. Studies allowing the tracing of the historical evolution of knowledge regarding the taxonomy of the genus *Giardia* were included, ranging from early morphological classifications to current genetic characterization methodologies.

Data Analysis and Extraction Criteria: Epidemiological and genetic data were evaluated at a global level, integrating the baseline results of a landmark worldwide review of molecular characterization studies of *Giardia* isolates obtained from humans up to 2021, alongside supplementary localized data published up to the year 2026.

Structural Analytical Framework: Although early epidemiological associations led international authorities to designate *Giardia duodenalis* as a major zoonotic pathogen, modern molecular epidemiology reveals a far more nuanced paradigm. Accumulating multilocus and genomic data demonstrate that most infections are host-restricted, indicating that anthroponotic cycles drive the vast majority of human cases. Nevertheless, critical ambiguities persist regarding the true frequency of cross-species spillover, the diagnostic challenge of differentiating passive cyst transit from active infection, and the clinical implications of assemblage-specific virulence. Clarifying these transmission dynamics is imperative to optimize public health strategies and refine interventions within the One Health framework. Here, we integrate genetic, clinical, and epidemiological evidence to delineate the precise boundaries and public health risks associated with *G. duodenalis* zoonotic transmission.

A structured search was executed across three primary electronic databases: PubMed/MEDLINE, Scopus, and Web of Science. The search covered literature published up to April 2026, utilizing combinations of the following Medical Subject Headings (MeSH) terms and keywords coupled with Boolean operators: (‘Giardia’ OR ‘giardiasis’) AND (‘zoonotic’ OR ‘zoonosis’ OR ‘cross-species transmission’) AND (‘assemblage’ OR ‘genotype’ OR ‘molecular epidemiology’) AND (‘animals’ OR ‘wildlife’ OR ‘livestock’ OR ‘pets’). Inclusion criteria were strictly limited to peer-reviewed original research articles, short communications, and authoritative reviews published in English that provided molecular or epidemiological evidence of *Giardia* transmission dynamics at the human–animal interface. Document types such as conference abstracts, doctoral dissertations, and non-peer-reviewed reports were excluded to maintain a high threshold of data reliability. Relevant studies were ultimately selected and synthesized to form the evidentiary basis of this review.

## 2. Evolution of Knowledge on the Taxonomy of the Genus *Giardia*

In general terms, the evolution of knowledge about the taxonomy of the genus *Giardia* has unfolded in two major stages: the first, marked by the description of as many species of the parasite as there were host species in which it was found, which culminates with a paradigmatic work on morphological aspects of the protozoan published by Filice in 1952 [14], which reduced the number of species in the genus to three; and the second, characterized by a better understanding of the genetic characteristics of *Giardia* species from different hosts, which is leading, once again, to the principles of the parasite’s specificity for the animal that hosts it and, consequently, to the description of a greater number of species within the genus [1,2]. These historical phases are examined in greater detail below.

### 2.1. From Host Specificity to Morphological Similarity

During the decades of the 1920s and 1930s, based on the differences in the species of their hosts (more than 40 according to some authors), numerous species of the genus *Giardia* were described [15]. Prominent examples from this period included designations such as *Giardia canis* from dogs, *Giardia bovis* from cattle, and *Giardia caprae* from goats. This practice led to an artificial inflation of the genus’s taxonomic diversity, as these isolates were later recognized as morphologically identical and ultimately synonymized under the *Giardia duodenalis* complex [15]. The idea of “one host, one parasite” completely ruled this age. A common assumption among parasitologists was that a protozoan must be a distinct parasite species if it was discovered in a new host species.

Simon, in 1922, used mainly size ratios, body proportions (length-to-width ratios), and the relative position/size of the nuclei under light microscopy to distinguish between the parasite found in humans and those found in mice, accepting the names *G. lamblia* and *G. muris* to designate the former and the latter, respectively [16].

In 1952, Filice published a detailed description of the morphology of the genus *Giardia* based on three distinct median body morphologies: claw-hammer shaped in *G. duodenalis*, small, rounded/granule-like in *G. muris* and long, slender, needle-like (or bow-shaped) in *G. agilis*. Only three species of the parasite were recognized: *G. duodenalis*, observed in humans and other mammals; *G. agilis*, found in amphibians; and *G. muris*, found in mice [14]. This reliance on purely structural taxonomy mirrored standard parasitological practices of the era. In fact, during this same period, genera like *Entamoeba* (e.g., separating *E. histolytica* from *E. coli*) and *Trypanosoma* were similarly undergoing massive visual reassessments based on biometrics and staining [3].

According to the taxonomic rationalization proposed by Filice, *G. duodenalis* could parasitize, in addition to humans, other species of domestic and wild animals. It is worth noting that *G. lamblia* (as well as *G. intestinalis*) is a junior synonym of *G. duodenalis*.

During the 1970s and 1980s, the hypothesis surrounding the zoonotic potential of *G. duodenalis* was considerably bolstered by accumulating epidemiological data. Specifically, investigation of waterborne giardiasis outbreaks frequently revealed tight spatio-temporal links between water source contamination and the detection of infected wild or domestic animals within the immediate vicinity of the affected watersheds [10,15].

The most notable outbreaks documented in the United States during this period included: Vail, Colorado (1978), considered one of the largest and most significant outbreaks in history, involving approximately 5000 people. The cause was the absence of a filter in the city’s water supply system. Camas, Washington (1976), an outbreak with 128 clinically confirmed cases. It was discovered that the contamination originated from streams, and this was the first major study to demonstrate that wildlife (specifically beavers) acted as a natural reservoir for the parasite. Rome, New York (1974), ~4800 cases helped confirm the direct link between tap water consumption and *Giardia* transmission, as the parasite’s cysts were found in local storage tanks [17]. In 1979, as mentioned earlier, both arguments led the WHO to draw the attention of the scientific community to the zoonotic potential of this species [10].

While early epidemiological correlations pointed to wild reservoirs (such as beavers) as primary contamination sources, conventional light microscopy lacked the resolution to distinguish human-infecting lineages from host-restricted animal genotypes [3]. This diagnostic impasse underscored the imperative to deploy advanced molecular tools—initially isoenzyme electrophoresis and subsequently polymerase chain reaction (PCR)—to definitively substantiate the dynamics of true zoonotic transmission.

### 2.2. From Morphological Similarity to Species Specificity

Filice’s proposal, although it promoted an important rationalization of the prevailing taxonomic chaos, was only a temporary solution. The phenotypic variability among *Giardia* ‘isolates’ from different hosts suggested the existence of other species, but the technological limitations of those years prevented proving it. The subsequent development of *in vitro* culture procedures, which allowed the axenic isolation and multiplication of *Giardia* isolates from some host species, became a significant step in this regard. Since then, it has been possible to yield parasites in sufficient quantity and homogeneity for further studies of phenotypic differentiation (for example, of ultrastructure and isoenzymes) and genetic characterization of species [18].

The next step, still using purely morphological criteria, was the study of ventrolateral flange and marginal plates of *Giardia* specimens from different host species. The higher resolution of electron microscopy made it possible to find additional morphological differences among some *Giardia* specimens obtained from some isolates, which led to the description of additional species. Currently, based mainly on microscopic features, epidemiological criteria and molecular biological markers, nine species are included within the genus *Giardia*: *G. duodenalis* (humans, numerous mammals), *G. muris* (primarily rodents of the family Muridae (mice and rats) as well as hamsters), *G. agilis* (amphibians), *G. psittaci* (psittacine birds), *G. microti* (preferentially infects rodents of the family Cricetidae, primarily voles (genera *Microtus* and *Myodes*) and muskrats (*Ondatra*) rodents), *G. ardeae* (herons), *G. peramelis* (quenda), *G. cricetidarum* (hamsters), and *G*. *varani* (water monitor) [13].

More recently, the application of biomolecular procedures (e.g., Polymerase Chain Reaction-Restriction Fragment Length Polymorphism (PCR-RFLP), Multilocus Sequence Typing (MLST) and Whole Genome Sequencing (WGS)) has allowed the genetic characterization of *Giardia* cysts obtained directly from fecal or environmental samples and thus solved the problem arising from the impossibility of cultivating *Giardia* specimens *in vitro* from some hosts [1,2,4,19]. The use of these procedures has made it possible to find genetic (assemblage) differences between *G. duodenalis* isolates from different host species (Table 1) [1,2,4,19].

The *bg* gene, *tpi* gene, and *gdh* gene are the three most critical nuclear genetic markers used for the molecular detection, assemblage typing, and evolutionary analysis of *G. duodenalis*. All three genes are highly polymorphic and sufficient to differentiate *Giardia* isolates. *bg* gene encodes β-giardin, an abundant structural protein specific to *Giardia* that works alongside microtubules and microribbons to help the parasite physically adhere to the host’s intestinal wall. It possesses a sequence diversity of roughly 5% between genotypes. *tpi* gene encodes a core, highly efficient glycolytic enzyme that catalyzes the reversible interconversion of dihydroxyacetone phosphate (DHAP) and D-glyceraldehyde-3-phosphate (G3P). This gene is vital to its metabolic survival. The *tpi* gene displays the highest degree of genetic heterogeneity among the standard markers. Finally, the *gdh* gene encodes an NADP-dependent enzyme crucial to *Giardia’s* anaerobic metabolism. It is a single-copy gene and its sequence shows intense ancient divergence, sharing high homology with eubacterial sequences [1,2,4,11,12].

These biomolecular findings have led to the description of *G. duodenalis* genetic assemblages which, validating the historical hypotheses of scholars of the subject in the first half of the 20th century, exhibit a high degree of host specificity [1,2,4].

## 3. *Giardia duodenalis*: A Species or a Complex of Them?

During the last four decades, a period that marks the beginning of modern molecular parasitology, studies based on a wide variety of clinical, epidemiological, phenotypic and genetic criteria have shown that *G. duodenalis* is not a uniform species. The application of molecular tools, such as PCR, MLST, and WGS, has revealed significant interspecies genetic variation (distinct genetic lineages), though they are traditionally classified as intra-species variations under a single umbrella name among *Giardia* isolates from different hosts [2].

Conventional, nested and quantitative PCR methods, using primers designed from the nucleotide sequences of genes that code for several polymorphic loci, have enabled the identification of eight assemblages (A to H) within this species [2,19,20]. The most common genotyping loci used for the molecular characterization of *G. duodenalis* have been *bg*, *tpi*, and *gdh* genes [2,4,21]. Conversely, while the highly conserved nature of the small subunit ribosomal RNA (*SSU rRNA*) gene renders it an exceptional marker for genus-level identification and broad assemblage screening, exclusive reliance on MLST frameworks introduces prominent analytical limitations. Chief among these is the high degree of allelic sequence heterozygosity (ASH), particularly widespread in assemblage B, which introduces overlapping nucleotide signals during Sanger sequencing and complicates unambiguous haplotyping [22]. Furthermore, contemporary genomic surveys frequently encounter locus incongruence or ‘assemblage swapping’ across different markers (e.g., *bg*, *gdh*, and *tpi*). This phenomenon, driven by genetic recombination or hidden mixed infections, underscores that single-locus or low-resolution MLST approaches can mask the true multi-clonal architecture of *Giardia* populations [14,22].

### 3.1. Molecular Characterization of Giardia Isolates Obtained from Humans

The molecular characterization of *Giardia* isolates obtained from humans allowed researchers to demonstrate that they align into one of two groupings [2,21,22]. These groups, both of widespread global distribution and also present in other mammal species, were named in 3 different ways: in Europe, as the Polish and Belgian groups; in North America, as groups 1/2 and 3; and in Australia, as assemblages A and B [19]. Of these names, the term assemblage has been the most used because it best reflects the fact that these groups include a genetically diverse collection of isolates that are not confined to a particular geographic location [19].

To resolve the historical ambiguity surrounding *Giardia duodenalis* nomenclature, the correlation between legacy, geographically isolated classification groups and modern molecular assemblages (A and B) must be explicitly delineated. Prior to the global standardization of the term ‘assemblage’ in Australia, European (Poland/Belgium) and North American investigators relied on distinct classification frameworks established via early isoenzyme, RFLP, and surface antigen analyses [19].

Specifically, modern assemblage A encompasses the historic North American Groups 1 and 2. At the sub-assemblage level, AI corresponds to the zoonotic Group 1 (isolated from both human and animal hosts), whereas AII maps directly to Group 2 (almost exclusively restricted to humans). In the European system, assemblage A aligns strictly with the Polish group. Conversely, modern assemblage B corresponds to North American Group 3, a historically recognized cluster characterized by high genetic diversity that is currently partitioned into sub-assemblages BIII and BIV. This same genetic population aligns exactly with the Belgian group within the legacy European framework [19].

In light of the growing consensus favoring the dissolution of the *G. duodenalis* complex into distinct species, several investigators have championed the reinstatement of specific binomial names. Under this multi-species framework, it has been proposed that assemblage A be formally designated as *Giardia lamblia*, whereas the genetically distinct assemblage B would be classified as *Giardia enterica* [1,2]. While this taxonomic split reflects the profound evolutionary distance separating both groups, the universal adoption of these names remains a subject of ongoing debate within the international nomenclature community.

Notably, evidence indicates that the genetic distance separating assemblages A and B exceeds that observed between distinct protozoan species [1]. Although this divergence suggests they could represent separate species, substantial genetic distance alone does not automatically denote speciation in asexual or recombining diplomonads without clear supporting biological, physiological, and reproductive boundaries [1]. However, the existence of genetic differences between isolates corresponding to each assemblage, which has led to the description of groupings within both, has made it advisable to wait for additional data for the definitive recognition that two species of the genus *Giardia* parasitize humans. Obviously, and as has occurred on the occasion of the redescription of species corresponding to other parasites, this taxonomic novelty would have important biomedical and epidemiological implications. Specifically, elevating these assemblages to distinct species status is underscored by documented variations in clinical virulence and drug sensitivity profiles. For instance, epidemiologic cohorts frequently associate assemblage B (*G. enterica*) with persistent diarrhea, bloating, and macro-nutritional malabsorption, whereas assemblage A (*G. lamblia*) often presents as acute, self-limiting infections or asymptomatic colonization [3,8]. Furthermore, from a therapeutic standpoint, *in vitro* and clinical isolates reveal marked discrepancies in susceptibility to first-line nitroimidazoles; assemblage B frequently displays higher tolerance or targeted resistance to metronidazol compared to assemblage A, rendering a unified clinical approach increasingly obsolete [4,21].

A worldwide review of molecular characterization studies of *Giardia* isolates obtained from humans up to 2021 revealed that assemblages A and B were present in 42.18% (2981/7067) and 52.51% (3711/7067) of the genotyped samples, respectively [20].

Although in much lower numbers, that work also documented canine-specific assemblages C and D in 0.53% (38/7067) of cases, and the felid-specific assemblage F in 0.09% (6/7067) [20]. However, when interpreting the detection of these characteristically host-specific animal genotypes (C, D, and F) in human fecal samples, a stringent cautionary note is warranted. In modern molecular epidemiology, such anomalous findings are rarely indicative of genuine, productive zoonotic colonization. Instead, they are predominantly attributed to the transient mechanical passage of non-replicating cysts following close contact with domestic pets, or alternatively, to minor PCR artefacts and cross-reactivity during low-resolution multi-locus sequencing assays [20]. Differentiating true clinical infection from passive intestinal transit remains a critical diagnostic challenge that requires careful epidemiological validation. The remaining 4.69% of the global datasets accounted for mixed infections characterized by the simultaneous presence of assemblages A + B (4.43%; 313/7067), alongside sporadic, transient spillover events involving livestock-specific assemblage E (0.24%; 17/7067) and rodent-specific assemblage G (0.02%; 1/7067) [20].

Assemblage A consists of *Giardia* isolates from humans and closely related animals. These isolates can be grouped into 3 sub-assemblages: AI, formed by *Giardia* organisms obtained from animals (farm, companion, and wild animals) and, to a lesser extent, from humans; AII, constituted exclusively by *Giardia* organisms detected in humans; and AIII, composed of *Giardia* organisms found in feces of wild ruminants [1,4,7,21]. A review of genotyping of *G. duodenalis* assemblage A isolates from humans identified 26.34% (552/2095), 71.65% (1501/2095), and 2.00% (42/2095) as classified in sub-assemblages AI, AII, and AIII, respectively [4].

Assemblage B comprises *Giardia* isolates that can be classified into two subgroups: sub-assemblage BIII, formed by *Giardia* organisms obtained from humans and other animals, and sub-assemblage BIV, apparently specific to humans [1,3]. Compared to assemblage A, the level of genetic diversity is higher among the isolates of assemblage B [1,3]. *Giardia duodenalis* sequences generated at the *gdh*, *bg* and *tpi* loci in studies conducted in geographical areas of high and low endemicity found that assemblage A grouped together in well-defined clusters (AI, AII and AIII) with appropriate reference sequences [23]. Nevertheless, although assemblage B sequences also formed a well-supported clade, sub-assemblage BIII and BIV sequences could not be segregated in completely independent clusters [23]. In line with it, in a sequencing study of *Giardia* organisms found in fecal samples from Mozambican children, the high level of genetic variability within assemblage B did not allow complete differentiation between subassemblages BIII and BIV and, consequently, 59% (132/222) of the total sequences analyzed were identified as ambiguous BIII/BIV results [24]. This is often due to allelic sequence heterozygosity (ASH) or mixed infections, which is an intrinsic feature of assemblage B. From a taxonomic perspective, the greater genetic distance separating many members of assemblage B suggests an earlier phylogenetic divergence. Furthermore, the elevated (ASH) and genetic diversity observed within assemblage B may stem from higher recombination rates, a larger effective population size, or ancestral hybridization [1].

### 3.2. Molecular Characterization of Giardia Isolates Obtained from Other Animals

Throughout the last three decades, six new assemblages of *G. duodenalis* (C to H) have been demonstrated in isolates or biological samples from numerous animals [1]. The *Giardia* isolates aligned in these assemblages are morphologically identical to those from humans, but the sequences of the genes encoding some molecules are different from those of assemblages A and B [1,2,3,4]. Two aspects characterize the *Giardia* organisms belonging to those assemblages:

- They are genetically more uniform than the *Giardia* isolates corresponding to assemblages A and B. No groups or subgroups are described within assemblages C to H [21,22].
- They exhibit greater host specificity than *Giardia* organisms corresponding to assemblages A and B [25]. Assemblages C and D are mainly found in canids [26]; assemblage E in hoofed mammals such as cattle, sheep, goats, and pigs [27]; assemblage F in felids [28]; assemblage G in rodents [26]; and assemblage H in seals [2].

Both genetic uniformity and host specificity strongly support the future recognition of assemblages C through H as distinct species within the genus *Giardia*. However, such taxonomic reclassifications will require comprehensive comparative genomics and formal ICZN validation, rather than relying solely on single- or multi-locus host association patterns.

Human infection with the host-adapted *G. duodenalis* assemblages C, D, E, and F has been described [2]. However, non-human infection with assemblages A (mainly subassemblage AI) and B (mainly subassemblage BIII) has been documented with more frequency [13,28]. The zoonotic potential of those findings, discussed in detail below, has received significant attention in recent years.

## 4. Evidence of Zoonotic Transmission

Epidemiological data have been used to give some support to the potential zoonotic transmission of human giardiasis. In several studies, close contact with farm, companion, and wild animals infected with *G. duodenalis* was considered a source for human giardiasis, reviewed in [2]. However, those works provided only circumstantial evidence on the possible occurrence of zoonotic transmission [2].

Molecular data have been useful in understanding the zoonotic potential of isolates in various animals [29]. Although *Giardia* organisms of assemblage A have been identified in biological samples of livestock, pets, rodents, and wild mammals, this genotype frequently accounts for less than 10% of *G. duodenalis* infections found in them [2]. Those animals are, in fact, mostly infected with host-adapted *G. duodenalis* assemblages, which are rarely found in humans [29]. Current molecular data indicate that assemblage A accounts for a lower global prevalence in human populations compared to assemblage B, rather than implying a reduced clinical severity or milder symptomatology. This lower epidemiological distribution underscores the dominance of assemblage B in driving persistent anthroponotic transmission networks across most endemic regions [20]. As humans are more commonly infected with assemblage B and there are differences in the distribution of assemblage A subtypes between humans and animals, increased attention has been directed to zoonotic transmission of assemblage B, which appears to be the dominant *G. duodenalis* genotype in a few groups of animals, such as beavers, muskrats, chinchillas, rabbits, horses, and nonhuman primates (reviewed in [29]). Some of those animals are aquatic or their fecal materials can be easily transported to water, thereby contributing to source water contamination with *G. duodenalis* assemblage B [29]. Nevertheless, the identification of host-adapted subpopulations within assemblage B has been hindered by inconsistent classifications of isolates across genetic loci because of ASH [2]. Overcoming this limitation requires further development of high-resolution typing tools.

### 4.1. Waterborne Transmission

Contamination of water sources for human use with *G. duodenalis* typically arises from anthropogenic wastewater influx, intensive agricultural and livestock practices, or the localized shedding of cysts by wild reservoirs inhabiting adjacent ecosystems [2,22]. For a long time, the detection of *Giardia* organisms morphologically identical to those of human origin in some hosts, mainly wild animals and cattle, has been the primary argument used to consider *G. duodenalis* a zoonotic microorganism [30,31].

Although wild animals, especially aquatic ones, are frequently infected with *Giardia*, there is limited evidence implicating them as primary contaminants of water sources [31]. Certainly, it is more likely that such animals become infected from waters contaminated with human fecal matter or even with the feces of domestic animals. Among all wild animals of concern, the strongest evidence of *G. duodenalis* zoonotic transmission came from molecular epidemiological characterizations of isolates from beavers in North America, where giardiasis is known as “beaver fever”. Beaver infection by assemblages A and B of *G. duodenalis* has been documented [4,29,31,32,33]. Data from recent comparative genomics analysis have supported their long-suspected role as an amplification or reservoir host for *G. duodenalis* in watersheds [34,35]. Nevertheless, the available evidence does not grant these animals an important role as a source of human disease. For instance, the possible effects on public health of the reintroduction of European beavers in Scotland were studied. It was observed that the risk of these animals having a significant impact on drinking water infection with *G. duodenalis* was low [31]. This marked discrepancy in public health risk between geographical regions is fundamentally rooted in host-specific baseline prevalences and assemblage dynamics. While North American beavers (*Castor canadensis*) maintain stable, endemic, and high-prevalence sylvatic cycles dominated by zoonotic assemblages A and B—thereby functioning as potent amplification hosts within watersheds—the European beaver (*Castor fiber*) exhibits an exceptionally low baseline infection rate. Furthermore, multi-locus typing of *C. fiber* in Europe predominantly yields non-zoonotic, host-adapted genotypes or transient, spillover infections (such as assemblage E) acquired from local livestock, rather than a self-sustaining zoonotic reservoir [31,34]. Acknowledging these evolutionary and ecological differences explains why the wild-host amplification risk remained negligible in the Scottish context compared to historical North American outbreaks.

Cattle, goats and sheep are predominantly infected with *Giardia* assemblage E isolates, with zoonotic assemblages A and B being infrequently detected [13]. It has been shown that calves infected with the assemblage A of *G. duodenalis* commonly excrete from 10^5^ to 10^6^ cysts per gram of feces [36]. This would mean that a small number of calves infected with the zoonotic assemblage A could represent a significant threat to public health (directly, for livestock handlers; and indirectly, as an important reservoir for waterborne giardiasis outbreaks). However, the available experimental and epidemiological evidence does not support the transmission of *Giardia* infection between humans and cattle. This host-adapted genotype consistently outcompetes zoonotic strains within the bovine gut, representing a clear manifestation of competitive exclusion. Longitudinal surveys indicate that up to 100% of livestock raised under natural field conditions become exclusively colonized by assemblage E within their first weeks of life, as its evolutionary adaptation to the ruminant intestinal niche effectively blocks the establishment of transient anthropogenic lineages [37].

Only under very particular circumstances, where *G. duodenalis* infection has not occurred previously and, consequently, the host-adapted genotype might be absent, does it seem possible to introduce and perpetuate a zoonotic genotype. For example, the results of a molecular epidemiological survey conducted in a remote national park in Uganda suggest that anthropogenic pressure led to the spillover of *G. duodenalis* into domestic cattle populations via spill-back infection. This finding indicates potential human introduction of *Giardia* infection to domestic cattle populations, explaining the presence of assemblage A in calves within that location [38].

The likelihood of dogs and cats serving as sources of waterborne contamination with potentially zoonotic *Giardia duodenalis* appears to be minimal. They are susceptible to infection by zoonotic assemblages of *G. duodenalis*, but the occurrence of events of water source contamination via domestic pet fecal runoff is highly improbable [30].

### 4.2. Direct Transmission

Dogs and cats provide emotional support and companionship, significantly impacting human daily lives. Consequently, they are the animals most frequently involved in the direct transmission of *G. duodenalis* infection to humans (and vice versa). Although the clinical consequences of *G. duodenalis* infection in dogs and cats appear to be minimal, the epidemiological significance of potential transmission to humans has received substantial attention in recent years [21].

During the second half of the 1990s, several studies led to the identification in dogs of *G. duodenalis* of the assemblage A [19]. Motivated by those first findings, some studies have shown infection by *Giardia* organisms of either zoonotic or host-specific assemblages in dogs [1,4,31,39]. For instance, a study conducted in Germany on fecal samples from dogs positive for *G. duodenalis* found that 60% corresponded to assemblage A, 12% to the dog-specific assemblages C and D, and the remaining 28% harbored mixed infections [40]. Similarly, a smaller number of studies have reported infection by *Giardia* organisms of either zoonotic or host-specific assemblages in cats [39]. For example, a study conducted in Mississippi and Alabama, USA, on 17 fecal samples from cats positive for *G. duodenalis* found that 6 were of assemblage A, and 11 of the cat-specific assemblage F [41]. However, as we mentioned before, such genotype findings did not confirm the zoonotic nature of the transmission. Trying to move in that direction, a cross-sectional molecular epidemiological survey investigated whether zoonotic disease transmission was occurring among humans and domestic dogs and cats sharing the same spatial and temporal setting in both rural and urban areas of the province of Álava, Spain. This study, which included 63 households with domestic cats and dogs, found no evidence that they were a significant reservoir for human infection [42].

Better evidence that zoonotic transmission could be taking place came with the results of two studies which examine the dynamics of *G. duodenalis* transmission in humans living in different environments: (i) in areas where the frequency of transmission of zoonotic and non-zoonotic assemblages is high, such as in Aboriginal communities in Australia, where dogs usually remain in packs, assemblage D is predominant [43], (ii) in areas where the frequency of transmission of zoonotic and non-zoonotic assemblages is lower, such as in tea-growing communities in the Assam region of India, where dogs regularly sleep with their owners and could coprophagy, only 20% of the dogs were infected, and notably, zoonotic assemblages—predominantly assemblage A—were exclusively detected. Apparently, this difference with respect to Aboriginal communities reflects a closer association between individual dogs and their owners in the tea-growing communities, and the frequency with which dogs are able to coprophagy in the latter [44].

The strongest evidence in favor of direct zoonotic transmission came from another study conducted in the tea-growing area of the Assam region, India. Traub et al., using multiple loci (*SSU rRNA*, *ef-1*, and *tpi*) for inferring genotypes of *Giardia* isolates, found the same assemblage in both people and dogs, not only in the same community but also in the same household. That is, zoonotic transmission was further supported by strong epidemiological evidence: the association between the presence of *G. duodenalis* infection in humans and the presence of a dog infected with *G. duodenalis* in the same household [11,30,45]. However, it is critical to note that high household concordance of identical assemblages within the same domestic setting does not definitively establish the direction of transmission. Such spatial and temporal overlapping cannot distinguish between true zoonotic spillover, anthroponotic reverse zoonosis (human-to-animal), or simultaneous exposure to a common external vehicle, such as contaminated water or food. To render these transmission dynamics watertight, contemporary molecular epidemiology must move beyond locus-specific consensus typing toward in-depth haplotype analyses, sub-assemblage discrimination, or WGS capable of resolving micro-evolutionary single-nucleotide polymorphisms (SNPs) to trace exact chain-of-infection trajectories [11,30].

## 5. The “One Health” Approach to *Giardia duodenalis* Zoonotic Transmission

**Figure 1:**
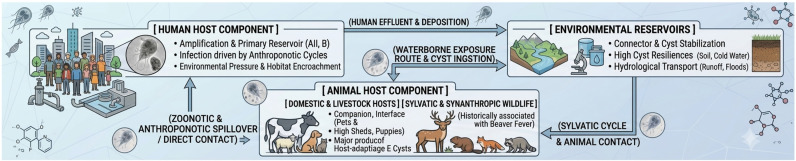
Schematic representation of the “One Health” triad in *Giardia duodenalis* transmission, illustrating the dynamic, closed cycle and specific interfaces between the Human Host, Animal Host (Domestic and Wildlife), and Environmental components. Mechanism-focused labels—such as “Anthroponotic Cycles”, “Waterborne Exposure Route”, “Fecal Shedding”, “Zoonotic spillover”—define the precise dynamics within this interconnected ecosystem.

The “One Health” approach provides the ideal framework for addressing the transmission dynamics of *Giardia duodenalis*, as this parasite seamlessly crosses boundaries between host species, urban centers, and natural ecosystems (Figure 1). The following sections outline how the three foundational pillars of the “One Health” triad—Human, Animal, and Environmental—interact within the giardiasis transmission cycle:

1. The Human Component: Host and Amplifier
  - Amplification and Reservoir: Humans act as both primary reservoirs and potent amplifiers of the parasite within urban and peri-urban environments.
  - Overcrowding and Sanitation: Deficiencies in personal hygiene, localized infrastructure, and inadequate municipal wastewater treatment directly facilitate the horizontal spread of the pathogen.
  - Anthroponotic Cycles: *G. duodenalis* assemblages A (primarily sub-assemblage AII) and B are the predominant drivers of human infection, effectively maintained through continuous person-to-person transmission pathways.
  - Anthropogenic Environmental Pressure: Human encroachment into wild habitats via ecotourism, deforestation, and agricultural expansion facilitates closer contact with wildlife, introducing human-adapted strains into sylvatic cycles, and vice versa.
2. The Animal Component: Domestic and Wildlife Reservoirs
  This pillar represents the epidemiological bridge connecting wild ecosystems, rural environments, and urban households.
  - Domestic Pets (Dogs and Cats): Companion animals frequently harbor host-specific assemblages C and D (dogs) or F (cats). However, due to close physical proximity and shared domestic environments with humans, they can acquire zoonotic assemblages A and B, participating in potential bidirectional spillover events.
  - Livestock (Cattle and Sheep): Young ruminants are major producers of host-adapted assemblage E cysts, but they can also excrete significant quantities of zoonotic assemblage A cysts. Inappropriate agricultural disposal or management of livestock manure directly contaminates pasture soils and adjacent surface waters.
  - Wildlife and Hidden Reservoirs: Wild animals serve as endemic reservoirs in natural ecosystems, complicating environmental control efforts. Semi-aquatic mammals, such as beavers and muskrats, are classic examples (historically linked to “beaver fever”), maintaining stable environmental parasite loads in pristine rivers and lakes.
  - Synanthropic Species: Wild animals adapted to urbanized areas (e.g., raccoons, rodents, and foxes) act as synanthropic reservoirs. By feeding on anthropogenic waste and defecating in residential or recreational public areas, they actively bridge the gap between sylvatic and urban transmission cycles.
3. The Environmental Component: The Vehicle of Dissemination
  The environment is not a passive backdrop; it functions as the central connective vehicle that stabilizes, preserves, and transports the parasite.
  - Cyst Resilience: The infectious cyst stage is highly resistant to adverse soil conditions, ambient osmotic pressure, and standard chlorine concentrations used in drinking water treatment. Cysts can remain viable and infectious for several months, particularly in cold water.
  - The Hydrological Cycle: Agricultural runoff, heavy rainfall, and flooding events mechanically transport fecal material from wild and domestic animals into municipal drinking water sources or irrigation systems used for ready-to-eat crops.

Ultimately, attempts to control and mitigate giardiasis through isolated medical interventions in human populations are insufficient. Achieving effective, long-term control necessitates integrated strategies that combine preventive veterinary medicine, strict livestock waste management, watershed protection, and the preservation of ecological balance within wild ecosystems.

## 6. Conclusions

While molecular evidence points to high host specificity among most *Giardia* strains—indicating that interspecies transmission is sporadic—this pathway remains a critical public health consideration for vulnerable populations, particularly young children and immunocompromised individuals. In these high-risk cohorts, even isolated zoonotic events can precipitate severe clinical complications, including chronic diarrhea, malabsorption, and failure to thrive, thereby highlighting the need for targeted preventive strategies.

Currently, research on the zoonotic transmission of *G. duodenalis* faces critical gaps, particularly regarding the ecological drivers of species jumps and the precise epidemiological impact on high-risk groups. To address these limitations, the development of high-resolution genotyping tools must be prioritized; taxonomy explicitly notes that resolving the *G. duodenalis* complex into distinct species remains an ongoing scientific debate requiring genomic-level validation. Methodologically, key challenges remain around the limited sensitivity of current diagnostic methods in detecting mixed infections, highlighting the need for longitudinal studies within a ‘One Health’ framework to enhance risk modeling and epidemiological surveillance.

## Figures and Tables

**Table 1:** Host specificity, zoonotic potential, and proposed taxonomic nomenclature for *Giardia duodenalis* assemblages.

Genotype/Assemblage	Proposed Species Name	Primary Mammalian Hosts	Zoonotic Potential & Public Health Risk	Epidemiological & Genetic Characteristics
Assemblage A Sub-assemblage AI Sub-assemblage AII Sub-assemblage AIII	*Giardia lamblia* *	Humans, non-human primates, domestic pets, livestock, wild mammals, and wild ruminants.	Low	AI: Broad host range; active zoonotic spillover. AII: Predominantly anthroponotic human transmission. AIII: Exclusively restricted to wild ruminants.
Assemblage B Sub-assemblages BIII/BIV	*Giardia enterica* *	Humans, non-human primates, beavers, muskrats, horses, and chinchillas.	Low	Predominantly anthroponotic cycles, characterized by pronounced allelic sequence heterozygosity (ASH) and high environmental persistence.
Assemblage C/D	*Giardia canis* *	Canids (dogs, wolves, coyotes).	Very Low	Strictly host-adapted; human cases are exceptionally rare and typically transient.
Assemblage E	*Giardia bovis* * (or *G. caprae*)	Hoofed mammals (cattle, sheep, goats, pigs, water buffalo).	Low	Livestock-restricted; exerts competitive exclusion over zoonotic strains in the bovine gut. Rare human spillover.
Assemblage F	*Giardia cati* *	Felids (domestic and wild cats).	Very Low	Highly host-restricted; negligible risk to human public health.
Assemblage G	*Giardia simondi* *	Rodents (rats, mice).	None documented	Strictly host-adapted endemic cycles within wild and peridomestic rodent populations.
Assemblage H	No formal proposal	Marine mammals (seals, sea lions).	None documented	Host-specific sylvatic cycles restricted to marine ecosystems.

* These taxonomic reinstatements/reclassifications remain informal proposals and have not been officially validated by the International Code of Zoological Nomenclature (ICZN). Overstating these as established species names is premature.

## Data Availability

No new data were created or analyzed in this study. Data sharing is not applicable to this article.

## Abbreviations

The following abbreviations are used in this manuscript:

WHO	World Health Organization
PCR	Polymerase Chain Reaction
MLST	Multi-locus Sequence Typing
WGS	Whole Genome Sequencing
SSU rRNA	Small Subunit Ribosomal RNA
ASH	Allelic Sequence Heterozygosity
ICZN	International Code of Zoological Nomenclature
